# The Effect of Tailored Short Message Service (SMS) on Physical Activity: Results from a Three-Component Randomized Controlled Lifestyle Intervention in Women with PCOS

**DOI:** 10.3390/jcm12072466

**Published:** 2023-03-23

**Authors:** Alexandra Dietz de Loos, Geranne Jiskoot, Rita van den Berg-Emons, Yvonne Louwers, Annemerle Beerthuizen, Jan van Busschbach, Joop Laven

**Affiliations:** 1 Division of Reproductive Endocrinology and Infertility, Department of Obstetrics and Gynaecology, Erasmus University Medical Center, 3015 GD Rotterdam, The Netherlands; 2 Department of Psychiatry, Section Medical Psychology and Psychotherapy, Erasmus University Medical Center, 3015 GD Rotterdam, The Netherlands; 3 Department of Rehabilitation Medicine, Erasmus University Medical Center, 3015 GD Rotterdam, The Netherlands

**Keywords:** PCOS, lifestyle intervention, three-component, Short Message Service, exercise, physical activity, aerobic capacity

## Abstract

This analysis of secondary outcome measures of a randomized controlled trial was conducted to study the effect of a one-year three-component (cognitive behavioural therapy, diet, exercise) lifestyle intervention (LSI), with or without additional Short Message Service (SMS) support, on physical activity and aerobic capacity in overweight or obese women with polycystic ovary syndrome (PCOS). Women diagnosed with PCOS and a BMI > 25 kg/m^2^ were randomly assigned to LSI with SMS support (SMS+, *n* = 60), LSI without SMS support (SMS−, *n* = 63) or care as usual (CAU, *n* = 60) in order to lose weight. Based on results from the International Physical Activity Questionnaire (IPAQ), we found a significant within-group increase after one year for SMS+ in the high physical activity category (+31%, *p* < 0.01) and sitting behaviour decreased (Δ −871 min/week, *p* < 0.01). Moreover, the peak cycle ergometer workload increased within SMS+ (Δ +10 watts, *p* < 0.01). The SMS+ group also demonstrated a significantly different increase in walking metabolic equivalent of task minutes (METmin)/week compared with CAU after one year (Δ 1106 METmin/week, *p* < 0.05). Apart from this increase in walking activity, no other between-group differences were found in this trial. Overall, based on within-group results, SMS support seemed to help with improving physical activity and aerobic capacity and decreasing sedentary behaviour.

## 1. Introduction

Polycystic ovary syndrome (PCOS), characterized by ovulatory dysfunction, hyperandrogenism and polycystic ovarian morphology, is currently the most common endocrine disorder in reproductive-aged women [1]. This endocrine disorder is often associated with overweightness and obesity [2]. Furthermore, other clinical problems in women with PCOS may include derangements in reproductive, mental or metabolic parameters. The severity of the clinically expressed PCOS phenotype in these women is in turn negatively associated with increasing body mass index [3,4], which indicates that treatment strategies should focus on weight management.

Physical activity (any bodily movement produced by skeletal muscles that requires energy expenditure) and structured exercise (activity requiring physical effort, carried out to sustain or improve health and fitness), deliver metabolic, cardiovascular and psychological health benefits in the general population [1,5,6,7,8]. Additionally, isometric strength training (placing tension on particular muscles without moving the surrounding joints) demonstrated positive effects on dynamic strength and sport-related performance [9]. By contrast, sedentary behaviour (activities during waking hours in a seated or reclined position with energy expenditure less than 1.5 times resting metabolic rate [10]) has a negative impact on health and is linked to all-cause mortality [11,12]. Improving physical activity is a common element in the process of weight management. There are contradictory results on physical activity levels in women with PCOS. One study found these to be lower in women with than without PCOS. In particular, overweight or obese women with PCOS were less prone to be aligned with physical activity recommendations for weight maintenance or weight loss [13,14]. On top of this, high sedentary behaviour was extremely prevalent in this particular group. Additionally, women with PCOS were found to have an impaired aerobic capacity [15,16]. However, another study concluded that physical activity levels did not differ between obese women with and without PCOS [17]. Nonetheless, physical activity has a positive effect on overall health. Therefore, with the knowledge that obese women with PCOS suffer from poor metabolic, reproductive and mental health, this population should be motivated to be more physically active, achieve weight loss and maintain a healthy lifestyle [13].

The PCOS guidelines recommend a multi-component lifestyle intervention, including diet, behavioural strategies and physical activity, to achieve and maintain healthy weight [1]. However, health care providers are still searching for strategies to motivate this particular population and improve adherence to healthy lifestyle choices [18]. For example, one could promote physical activity by focusing on daily activities such as movement during transportation, work, leisure time or household and gardening chores when considering women’s individual and family routines as well as cultural preferences [1]. Furthermore, eHealth, the use of information and communication technology to improve health, has demonstrated to have the potential to effectively promote physical activity in adults with obesity [19]. Mobile health options such as text messages through the Short Message Service (SMS) may be used for this purpose [20]. Where SMS support is given, tailored text messages appear to be more effective than generic ones in the general population [21,22]. However, the evidence on changes in physical activity resulting from motivational strategies such as SMS support in addition to a lifestyle intervention is still limited in women with PCOS.

We previously performed a randomized controlled one-year multidisciplinary lifestyle intervention aimed at changing cognitions and dietary habits and encouraging and promoting physical activity [23]. Half of the participants allocated to this three-component lifestyle intervention also received additional SMS support. The control group received care as usual, which consisted of advice to lose weight through methods of their own choosing. The primary outcome measure, weight loss, was achieved more in the lifestyle intervention groups and especially in the group with SMS support. Moreover, the chance of achieving a 5% weights loss was 7.0 times greater in the lifestyle intervention groups than the care as usual group [24]. The current study in the same cohort focused on the effect of the lifestyle intervention, with or without SMS support, on weekly physical activity levels when compared with care as usual. We hypothesized that physical activity levels increased in those women who received the three-component lifestyle intervention and that tailored SMS support might have amplified these results. Additionally, changes in aerobic capacity were also evaluated within the lifestyle intervention groups.

## 2. Materials and Methods

### 2.1. Trial Design

The PCOS lifestyle study was a randomized controlled trial (RCT) performed between August 2010 and March 2016. Women were included within the division of Reproductive Endocrinology and Infertility of the Department of Obstetrics and Gynaecology, at the Erasmus University Medical Centre, the Netherlands. The following three groups were compared: (1) one-year three-component lifestyle intervention with SMS support (SMS+), (2) one-year three-component lifestyle intervention without SMS support (SMS−) and (3) one-year care as usual (CAU). Data were collected every three months from baseline up to and including one year. The study protocol was published previously [23]. This RCT was approved by the Medical Research Ethics Committee of the Erasmus MC in Rotterdam (MEC2008-337) and registered with the clinical trial number NTR2450 (www.trialsearch.who.int (accessed on 1 February 2023)).

### 2.2. Participants

Women were included who were actively trying to become pregnant, had a body mass index (BMI) > 25 kg/m², were between 18 and 38 years of age and had a diagnosis of PCOS according to the Rotterdam 2003 consensus criteria [25]. Exclusion criteria comprised inadequate command of the Dutch language, severe mental illness, obesity due to another somatic cause, androgen excess caused by adrenal diseases or ovarian tumours and other malformations of the internal genitalia. All participants provided written informed consent. The sample size calculation was based on a notable difference in weight as the primary outcome measure of this RCT. A minimum of 60 participants was needed in each group, when accounting for an expected dropout proportion of 40%. Randomisation of participants was in a 1:1:1 ratio to one of the three groups with the use of a computer-generated random numbers table, which was executed by a research nurse who was not involved in the study.

### 2.3. Three-Component Lifestyle Intervention (LSI) and Control Group (CAU)

The three-component lifestyle intervention for both the SMS+ and SMS− groups consisted of twenty 2.5 h group meetings over the one-year period that covered the following topics: (1) cognitive behavioural therapy (CBT), (2) normo-caloric diet and (3) physical activity. The first 1.5 h of each group meeting was supervised by a mental health professional and a dietician. CBT techniques were used to create awareness and to restructure dysfunctional thoughts about, e.g., self-esteem and weight (loss). Furthermore, dietary advice was discussed as recommended by the ‘Dutch Food Guide’ [26]. The last hour of each session focused on physical activity and was supervised by two physical therapists. During each session, different sports and exercises were performed to encourage participants to try new forms of physical exercise. Furthermore, participants were also encouraged to increase their general physical activity during their daily routine. Recommendations were based on the Global Recommendations for Physical Activity by the World Health Organization [27]. These recommendations included: (1) five days of moderate physical activity for thirty min each day, (2) vigorous exercise one to three days a week (at least twenty min per session) and (3) perform eight to ten muscle-strengthening activities involving major muscle groups twice a week. Every 3 months, participants discussed their improvements and pitfalls with the psychologist, dietician and physical therapist. 

After three months, the SMS+ group received SMS support in addition to the lifestyle intervention program. This group sent weekly self-monitored information regarding their diet, physical activity and emotions by SMS. A semi-automated software program returned patient-tailored SMS feedback to encourage positive behaviour. Additionally, two messages per week were sent addressing eating behaviour and physical activity; see Table 1.

In order to get acquainted with the program, we tested the lifestyle intervention in a pilot group (*n* = 26), of which the data were not included in the final analyses.

The control group received care as usual (CAU) as provided by health care professionals of our department for any woman with PCOS, excess weight and a wish to become pregnant. Their treating physician discussed the risk of excess weight for both mother and child and the relationship between overweightness and infertility. Subsequently, weight loss was encouraged by publicly available services such as visiting a dietician or gym.

### 2.4. Clinical and Endocrine Assessments

Participants of all three groups (SMS+, SMS− and CAU) received five standardized assessments every three months from baseline up to and including one year. These included general medical, obstetric and family history, physical measurements (height, weight, BMI (kg/m²), waist and hip circumference, blood pressure), transvaginal ultrasound (probe < 8 MHz) and an extensive endocrine assessment on fasting blood samples. Additionally, in order to monitor physical activity behaviour, all participants filled in the International Physical Activity Questionnaire (IPAQ) Long Form [28] at the above-mentioned three-monthly evaluation moments. Furthermore, a maximal cycle ergometer test was performed in the SMS+ and SMS− groups to evaluate changes in aerobic capacity. The CAU group did not perform the maximal cycle ergometer test intentionally, in order not to perform any form of intervention in this control group.

### 2.5. Outcome Measures

The primary outcome measure included the change in physical activity category (low, moderate, high) between and within all three groups over the course of the study period from baseline up to and including one year. These data were retrieved from the international physical activity questionnaires. Secondary outcome measures included changes in total weekly physical activity (metabolic equivalent of task minutes (METmin)/week), further subdivided per domain (work, transportation, household activities, leisure time (METmin/week)) and intensity (walking, moderate, vigorous (METmin/week)). Changes in sedentary behaviour (minutes/week) were also analysed. Furthermore, aerobic capacity within (only) the lifestyle intervention groups were evaluated and expressed as the achieved peak load (watt) resulting from the maximal cycle ergometer test. 

#### 2.5.1. International Physical Activity Questionnaire (IPAQ)

The IPAQ assesses the frequency, duration and intensity of physical activity in the course of the previous week and covers the following four domains: (1) at work, (2) during transportation, (3) during household activities and (4) during leisure time. The intensity of these various activities can be represented in metabolic equivalents (METs), which express energy expenditure in multiples of resting energy cost [29]. According to standardized procedures, time and days per activity and intensity were converted to MET minute/week scores by calculating METs x days x daily time. One minute of moderate household activities comprises 3.0 METs, walking 3.3 METs, general moderate intensity activities 4.0 METs, vigorous yard work 5.5 METs, cycling 6.0 METs and vigorous intensity activities 8.0 METS. Sedentary behaviour is also evaluated as an extra domain, which is expressed in minutes/week. Subsequently, subjects can be divided into three different physical activity categories:

1. Low: no activity is reported or some activity is reported but not enough to meet categories ‘moderate or high’. These women reported activity equivalent to less than 600 METmin/week.
2. Moderate: These women reported 3 or more days of vigorous activity of at least 20 min per day, 5 or more days of moderate-intensity activity and/or walking of at least 30 min per day, or 5 or more days of any combination of walking, moderate-intensity or vigorous-intensity activities equivalent to at least 600 METmin/week.
3. High: These women reported vigorous-intensity activity on at least 3 days equivalent to at least 1500 METmin/week or 7 or more days of any combination of walking, moderate- or vigorous-intensity activities equivalent to at least 3000 METmin/week [28,29].

#### 2.5.2. Maximal Cycle Ergometer Test

Before the start of every test we screened participants for cardiac and/or pulmonary contraindications with the Physical Activity Readiness Questionnaire (PAR-Q) [30]. Participants performed a standard ramp protocol on a cycle ergometer starting with a 5 min warm-up (20 watt) followed by an increase in load with 10, 15 or 20 watt every minute, based on the level of the participant. Participants must keep up a speed of 60 to 80 revolutions per minute. The test endpoint was a decrease of 15 revolutions per minute; at this point the peak load (watt), peak heart rate (beats per minute (BPM)) and modified Borg scale were evaluated. A maximum effort was defined as achieving an arbitrary 85% of the predicted maximum heart rate [31]. The predicted maximum heart rate was calculated with the use of Tanaka’s equation (maximum heart rate: (208 − (0.7 × age))) [32]. The modified Borg scale provides insight into the subjectively perceived effort level and ranges from 0 (no effort at all) to 10 (maximum exhaustion) [33]. A measurement in which the participant did not perform a maximum effort was excluded from the analyses. 

### 2.6. Statistical Methods

Physical activity category, weekly METs and sedentary behaviour minutes from the IPAQ responses were calculated according to standardized procedures [29]. Data distribution was evaluated using the Kolmogorov–Smirnov test. Baseline primary and secondary outcome measures were displayed as mean (standard deviation) in case of a normal distribution or as median (interquartile range (IQR)) in case of a non-normal distribution for continuous variables and as n (%) for categorical variables. Within-group and between-group differences over time were analysed with multilevel linear or logistic regression analyses for continuous and categorical variables, respectively. The reason being that mixed modelling is a preferred method when datasets have missing data and unbalanced time-points [34]. The model contained two levels comprising the participants and their repeated measures. Furthermore, the study group, logarithmic time and interactions were included as independent variables. In case of a non-normal distribution, we performed a bootstrap procedure with 10,000 samples in order to fulfil the assumption of normality for the multilevel regression analyses. The estimates of the models were displayed as means for multilevel linear regression analyses and as percentages for multilevel logistic regression analyses. Statistical significance was defined as *p* < 0.05. IBM SPSS statistics version 27.0 was used for multilevel linear analyses including the bootstrap procedure. SAS version 9.4 (SAS Institute Inc., Cary, NC, USA) was used for multilevel logistic regression analyses.

## 3. Results

**Figure 1:**
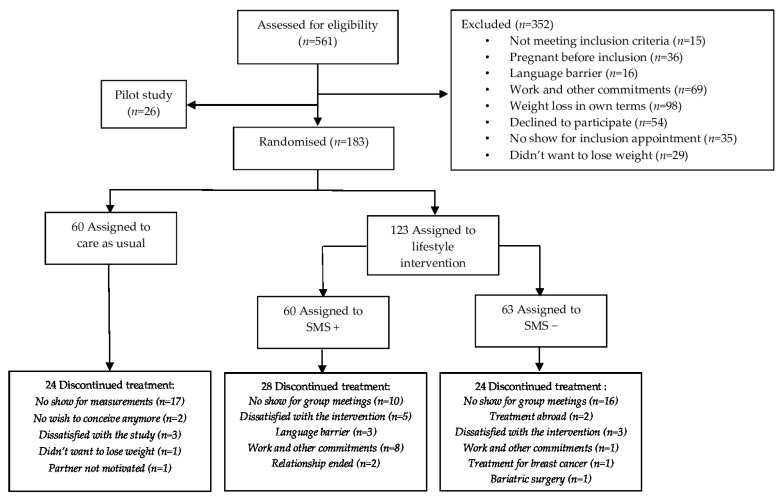
CONSORT flowchart.

For this RCT, we identified 561 eligible women between August 2010 and March 2016. Of these women, 352 were excluded for reasons further specified in Figure 1, and 26 women participated in the pilot study, which was not included in the final analysis. Eventually, 183 women were allocated to SMS+ (*n* = 60), SMS− (*n* = 63) or CAU (*n* = 60) and had a median age of 29 years (IQR 26–32) and median BMI of 32.8 kg/m^2^ (IQR 30.1–36.1). At baseline, only a small proportion of the participants were classified into the low physical activity category, ranging between 4.4 and 12.2% for all groups. The proportions of participants in the moderate and high physical activity categories ranged from 24.4 to 35.6% and from 60.9 to 63.4%, respectively; differences were all non-significant. Walking METmin/week was significantly different at baseline between the SMS+ (792 METmin/week) and CAU groups (1931 METmin/week) (*p* = 0.027) but not when compared with the SMS− group (1148 METmin/week). However, total physical activity METmin/week was similar between the groups, with 3834 (2007–5567), 3911 (2084–6555) and 3960 (1973–8573) for SMS+, SMS− and CAU, respectively; see Table 2.

### 3.1. Changes in Low, Moderate and High Physical Activity Categories Estimates

**Figure 2:**
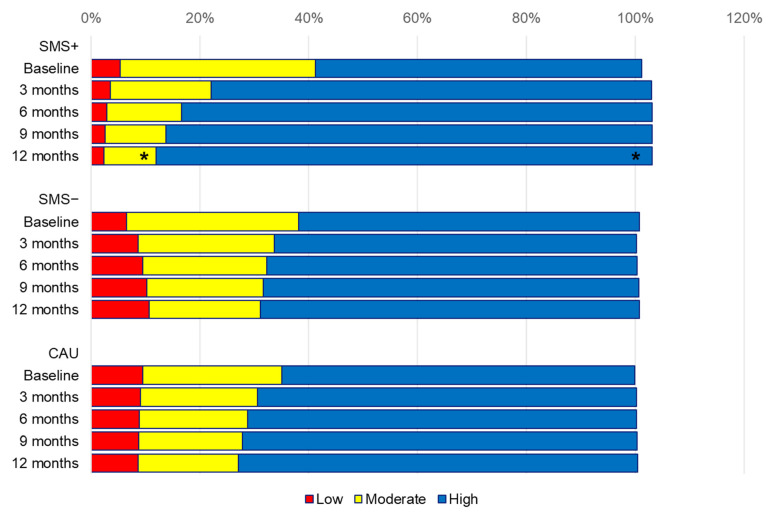
Changes in physical activity category estimates over time. Note: differences were tested with multilevel logistic regression analyses. * indicates significant within-group differences compared with baseline (*p* < 0.05). SMS+, lifestyle intervention with SMS support; SMS−, lifestyle intervention without SMS support; CAU, care as usual.

Remarkably, the biggest and statistically significant changes within the high, moderate and low physical activity categories were observed within the SMS+ group. There was a within-group increase of 31.0% (from 60.0% to 91.1%) in the high physical activity category over 12 months (*p* = 0.007) and a within-group decrease in moderate physical activity category (from 35.8% to 9.6%, Δ −26.1% within 12 months, *p* = 0.018). The low physical activity category within SMS+ did not change significantly (from 5.4% to 2.4%, Δ −3.0% within 12 months, *p* = 0.358). Within the SMS− group these differences were less prominent, with changes from 6.5% to 10.7% (Δ 4.2% within 12 months, *p* = 0.443) within the low category, 31.6% to 20.4% (Δ −11.3%, *p* = 0.251) within the moderate category and 62.6% to 69.6% (Δ 7.0% within 12 months, *p* = 0.515) within the high category. Moreover, for the CAU group there were changes from 9.5% to 8.7% (Δ −0.7% within 12 months, *p* = 0.917) in the low category, 25.6% to 18.4% (Δ −7.3%, *p* = 0.453) within the moderate category and 64.8% to 73.3% (Δ 8.4% within 12 months, *p* = 0.442) within the high category; see Figure 2. We did not observe any statistically significant between-group differences for changes in physical activity categories; see Table 3.

### 3.2. Physical Activity METminutes Estimates after 12 Months

**Figure 3:**
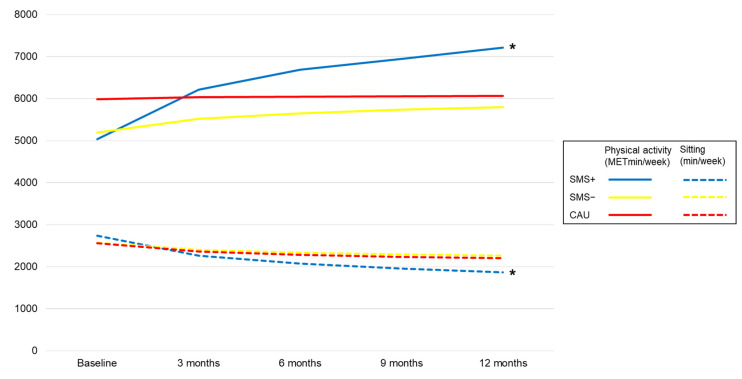
Changes in total physical activity METminutes and sitting behaviour minutes over time. Note: differences were tested with multilevel linear regression analyses, combined with a bootstrap procedure in case of a non-normal distribution. * indicates significant within-group differences (*p* < 0.05). MET, metabolic equivalent of task; min, minutes; SMS+, lifestyle intervention with SMS support; SMS−, lifestyle intervention without SMS support; CAU, care as usual.

Total physical activity METmin increased significantly within the SMS+ group, with 2175 METmin/week (*p* = 0.043), and non-significantly within the SMS− and CAU groups, with 610 METmin/week (*p* = 0.460) and 80 METmin/week (*p* = 0.944), respectively; see Figure 3. Between-group differences for total physical activity were non-significant. With regard to the different physical activity intensities, we observed a statistically significant higher increase in walking METmin/week within the timeframe of 12 months in the SMS+ group (from 1404 METmin/week to 2057 METmin/week) compared with the CAU group (from 2131 METmin/week to 1677 METmin/week) (*p* = 0.047, Δ 1106 METmin/week). Further details on estimated within-group and between-group physical activity changes within the different domains and for the different intensities are presented in Table 3 and Table 4. 

### 3.3. Sedentary Behaviour Estimates after 12 Months

Sedentary behaviour decreased significantly within SMS+ from 2735 min/week at baseline to 1864 min/week at 12 months (Δ −871 min/week, *p* = 0.005). Additionally, a non-significant decrease was observed within SMS−, from 2563 min/week at baseline to 2257 min/week at 12 months (Δ −306 min/week, *p* = 0.183), and within CAU, from 2559 min/week at baseline to 2198 min/week at 12 months (Δ −361 min/week, *p* = 0.157); see Table 4. Between-group differences with regard to sitting minutes were non-significant; see Table 3.

### 3.4. Aerobic Capacity Estimates after 12 Months within SMS+ and SMS−

We observed a significant increase in peak load resulting from the maximal cycle ergometer test within SMS+ from 177 watts at baseline to 187 watts at 12 months (Δ 10 watts (+5.5%) within 12 months, *p* = 0.005). For SMS−, this was 168 watts at baseline and 170 watts at 12 months (Δ 3 watts (+1.6%) within 12 months, *p* = 0.102). This was non-significant between the two groups (*p* = 0.222). Participants achieved on average 92–93% of the predicted maximum heart rate, which remained stable over the course of the study. The number of participants who delivered a maximum performance according to the pre-specified cut-off of ≥85% of the predicted maximum heart rate is further specified in Table 5.

## 4. Discussion

This randomized controlled study reports on physical activity outcomes following a three-component lifestyle intervention with or without additional SMS support. Apart from an increase in walking METmin/week in the SMS+ group compared with the CAU group after one year, we did not observe any other statistically significant between-group differences. However, the SMS+ group was successful at improving categories of self-reported physical activity and also demonstrated a statistically significant positive within-group effect on aerobic capacity and decreased weekly sitting minutes.

Other lifestyle interventions have described positive health benefits as a result of increased physical activity behaviour. Modest increases in step count were associated with reduced levels of inflammatory markers in women with PCOS [35]. Both high-intensity interval training (HIIT) and continuous aerobic exercise training have shown to improve reproductive function [36], anthropometrics and some cardiometabolic health markers [37,38]. However, HIIT has shown to offer greater improvements in aerobic capacity, insulin sensitivity and menstrual cyclicity and larger reductions in hyperandrogenism compared with moderate intensity training [39]. In the end, a recent meta-analysis concluded that improvements in health outcomes were more dependent on exercise intensity rather than dose [40]. However, especially with regard to adherence to a lifestyle intervention in this population, one should keep in mind an individual’s personal and cultural preferences when composing an exercise program in order to make it a sustainable lifestyle change. This may sometimes mean that health care providers should focus more on increasing general daily physical activity rather that promoting vigorous exercise.

Weekly sitting minutes decreased significantly within the SMS+ group during our one-year lifestyle intervention. Sedentary behaviour is extremely prevalent in the PCOS population [13], and positive associations were found between increased sitting time and weight gain [14,41], as well as PCOS symptom severity [42]. In the general population, sedentary behaviour is linked to all-cause mortality and adverse health impacts [11,12]. Therefore, one of the most important aspects should be to diminish sedentary behaviour in women with PCOS who struggle with weight loss or weight maintenance. 

The lifestyle intervention with SMS support demonstrated a statistically significant within-group increase in peak workload over the course of the study. However, the clinical relevance of the magnitude of this finding can be questioned. Notable improvements in aerobic capacity are generally to be expected following an increase in moderate and, especially, vigorous exercise [39,43]. An explanation for the modest improvements could be that one of the main goals of our lifestyle program was to encourage the implementation of a combination of moderate, vigorous and muscle-strengthening activities in the participant’s daily routine [23,27] and was therefore not designed as an intense, solely high-intensity exercise intervention. There are no studies that clearly define the clinical relevance of changes in peak workload or IPAQ responses in women with PCOS and excess weight. However, evidence does exists on the effect of weight loss and favourable changes in aerobic capacity [44]. Around 85.7% of the women in the SMS+ group achieved >5% weight loss [24], suggesting that the improvements in body weight might have positively impacted the results on peak workload. Furthermore, the observed positive changes to the high category at least indicates an increase in general weekly physical activity. Walking METmin/week improved more in the SMS+ group, corresponding to an increase of almost 30 min daily walking activity. Taking more steps per day has been found to be associated with a progressively lower risk of all-cause mortality in the general population [45]. Moreover, the decrease in sitting behaviour minutes, which in the SMS+ group amounted to several hours a day, may also be seen as a significant improvement. One could hypothesize that the above-mentioned findings do count as clinically relevant in this population of women with PCOS in which lifestyle habits are known to be difficult to improve [46].

A strength of our study was the use of tailored SMS in order to encourage and reinforce positive behavioural changes and increase physical activity. Although the PCOS guidelines recommend considering the use of mobile health applications for this purpose, limited evidence is available on the effectiveness of this method. In general, studies have suggested that the use of mobile technology for health promotion might be effective in improving long-term health-related outcomes [47,48]. Recently, a study concluded that a mobile health application, in addition to a lifestyle modification program, could decrease BMI, waist circumference, anxiety and depression and improve exercise and diet adherence in patients with PCOS in the long term [49]. Furthermore, another mobile health application called ‘AskPCOS’ has been recently developed in response to the specific needs of women with PCOS [50,51]. These are all indications that the use of supporting mobile health technology has a positive effect on behavioural changes and should be used to motivate adherence to a healthy lifestyle in the PCOS population. 

A limitation of the study is recall bias for weekly physical activity measured with IPAQ, which is a common problem in retrospective assessment with questionnaires. Self-reporting can cause over- and underestimation of weekly physical activity that may bias the results [52]. However, the IPAQ is an internationally used questionnaire with acceptable measurement properties and is at least as good as other established self-reporting methods [28]. In order to address the above-mentioned limitations, specific rules for processing data were applied according to the IPAQ protocol [29]. Nonetheless, the IPAQ data provide a good reflection of the participant’s weekly activities. Future studies should consider using devices such as an accelerometer or pedometer in order to objectively measure physical activity. Furthermore, when interpreting the results, one should keep in mind that this randomized controlled trial was powered on weight loss (primary outcome) and not on physical activity [23]. Additionally, the preferred assessment of aerobic capacity is measuring the maximum amount of oxygen uptake during exercise (VO_2_max) [53], which can be conducted using an open-circuit spirometry method. By measuring the gas exchange, the oxygen demands of the skeletal muscles during maximal physical exercise give a reflection of the peak capacity of the participant’s cardiovascular and pulmonary systems [54]. Open-circuit spirometry was not performed in our study population. However, VO_2_max is closely related to exercise workload. Therefore, the interpretation of the results of these two outcomes are comparable, although conclusions should be interpreted with caution. Finally, one could also interpret the absence of maximum cycle ergometer tests in the CAU group as a limitation. However, this was implemented intentionally because any form of interference could have influenced the control group’s actions. A recurrent maximal cycle ergometer test is not in line with care as usual and therefore could have impaired the results from the control group. 

## 5. Conclusions

Apart from an increase in walking activity in SMS+, no other between-group differences were found in this one-year three-component lifestyle intervention. However, based on within-group results, additional SMS support seemed superior in improving physical activity and aerobic capacity and decreasing sedentary behaviour in overweight and obese women with PCOS and a wish to become pregnant. Future adequately powered studies should be performed in order to confirm this positive tendency for eHealth options in the promotion of a physically active lifestyle.

## Figures and Tables

**Table 1:** Examples of text messages focused on physical activity.

Did you know that cleaning the house is also a moment of exercise? You can burn up to 140 calories in an hour!
Nordic walking is a fun form of walking that burns extra calories. Maybe it’s something for you?
Take the stairs one extra time every day this week. Your goal doesn’t have to be big, think of something small that you can change.
Vacuuming is also a form of exercise! Start cleaning this weekend!
Challenge: if you encounter an elevator this week, take the stairs!
Go for a walk during your break from work!
Household chores are also a form of exercise! While scrubbing the floor you can burn about 140 calories (per 60 min).
Try to go swimming with a friend this week. That will be fun!
Did you stick to the exercise standard of 30 min a day this week?
Did you know that exercise helps against fatigue and negative feelings?

**Table 2:** Baseline characteristics.

	Lifestyle Intervention				Care as Usual	
	SMS+ *n* = 60	Missing Values	SMS− *n* = 63	Missing Values	*n* = 60	Missing Values
	n (%)	n	n (%)	n	n (%)	n
Nulliparous	47 (79.7)	1	47 (75.8)	1	44 (75.9)	2
Caucasian	30 (50.0)	-	21 (35.0)	3	25 (42.4)	1
Smoking	13 (21.7)	-	11 (17.7)	1	14 (23.7)	1
Alcohol consumption	12 (20.0)	-	15 (24.2)	1	19 (32.2)	1
Education						
Low	5 (8.3)	-	5 (8.2)	2	8 (14.3)	4
Intermediate	33 (55.0)	-	34 (55.7)	2	35 (62.5)	4
High	22 (36.7)	-	22 (36.1)	2	13 (23.2)	4
IPAQ physical activity category	*n* = 45		*n* = 46		*n* = 41	
Low	2 (4.4)		3 (6.5)		5 (12.2)	
Moderate	16 (35.6)		15 (32.6)		10 (24.4)	
High	27 (61.4)		28 (60.9)		26 (63.4)	
	Median (IQR)	Missing values n	Median (IQR)	Missing values n	Median (IQR)	Missing values n
Age (year)	28 (26–32)	-	30 (27–33)	1	28 (26–32)	-
Weight (kg)	95 (85–106)	-	89 (80–104)	1	84 (79–97)	-
BMI (kg/m²)	33.5 (30.9–37.1)	-	33.6 (30.4–36.0)	1	30.6 (29.3–34.3)	-
Waist (cm)	102 (94–110)	4	100 (93–107)	4	96 (89–109)	1
IPAQ	*n* = 46		*n* = 47		*n* = 43	
Walking (METmin/week)	792 (330–2112)		1148 (446–2153)		1931 (512–4158)	
Moderate (METmin/week)	1935 (686–4447)		2160 (1050–4187)		1350 (720–3300)	
Vigorous (METmin/week)	960 (240–3840)		1096 (380–3540)		1440 (520–5280)	
Total physical activity (METmin/week)	3834 (2007–5567)		3911 (2084–6555)		3960 (1973–8573)	
Sitting (min/week)	2520 (1710–3630)		2730 (1725–3240)		2865 (1725–3360)	
Maximum cycle ergometer test	*n* = 31		*n* = 23			
Peak load	179 (148–210)		166 (134–208)		-	
Peak heart rate	173 (170–181)		168 (162–178)		-	
mBorg	7 (4–7)		6 (5–8)		**-**	

Note: Values are displayed as numbers (percentages) or as medians (interquartile range). Abbreviations: SMS+, lifestyle intervention with SMS support; SMS−, lifestyle intervention without SMS support; IQR, interquartile range; IPAQ, international physical activity questionnaire; BMI, body mass index; MET, metabolic equivalent of task; mBorg, modified Borg (rating of perceived exertion scale).

**Table 3:** Differences in physical activity categories and METmin/week between study groups at 12 months.

	SMS+ vs. CAU Difference	*p* Value	SMS− vs. CAU Difference	*p* Value	SMS+ vs. SMS− Difference	*p* Value
Category **%**						
Low	−2.2	0.312	4.9	0.543	−7.1	0.232
Moderate	−18.9	0.182	−4.0	0.823	−14.9	0.220
High	22.6	0.079	−1.5	0.922	24.0	0.060
Domains METmin/week						
Work	1574	0.293	1615	0.318	−42	0.981
Transport	−7	0.952	259	0.635	−266	0.479
Domestic and garden	−776	0.145	−264	0.665	−512	0.330
Leisure	547	0.502	−103	0.883	650	0.298
Intensity METmin/week						
Walking	1106	**0.047**	403	0.421	703	0.134
Moderate	−645	0.351	−508	0.417	−138	0.833
Vigorous	622	0.634	293	0.824	329	0.797
Total physical activity	2095	0.195	530	0.195	1565	0.243
Sedentary behaviour min/week						
Total sitting	−510	0.172	55	0.858	−565	0.141

Note: Differences were tested with multilevel logistic regression analyses for categorical variables, and with multilevel linear regression analyses for continuous variables, combined with a bootstrap procedure in case of a non-normal distribution. Boldface indicates significant difference (*p* < 0.05). Abbreviations: MET, metabolic equivalent of task; SMS+, lifestyle intervention with SMS support; SMS−, lifestyle intervention without SMS support; CAU, care as usual.

**Table 4:** Within-group changes in METmin/week from baseline to 12 months.

IPAQ Responses	Group	Baseline	3 Months	6 Months	9 Months	12 Months		
n	SMS+	46	21	10	8	5		
n	SMS−	47	29	22	17	14		
n	CAU	43	21	28	21	11		
Domains METmin/week	Group	Baseline	3 months	6 months	9 months	12 months	Change	*p* value within
Work	SMS+	3704	3845	3902	3938	3964	260	0.858
	SMS−	3428	3591	3656	3698	3729	302	0.823
	CAU	5047	4337	4050	3867	3733	−1313	0.200
Transport	SMS+	1203	1169	1155	1147	1140	−63	0.826
	SMS−	987	1097	1141	1169	1190	203	0.421
	CAU	1217	1187	1174	1167	1161	−56	0.904
Domestic and garden	SMS+	1633	1392	1295	1233	1187	−446	0.251
	SMS−	1531	1567	1581	1590	1597	66	0.853
	CAU	1446	1624	1697	1743	1776	331	0.220
Leisure	SMS+	1348	1783	1959	2071	2153	805	0.132
	SMS−	1393	1477	1510	1532	1548	155	0.639
	CAU	1481	1620	1677	1713	1739	258	0.661
Intensity METmin/week	Group	Baseline	3 months	6 months	9 months	12 months	Change	*p* value within
Walking	SMS+	1404	1757	1899	1990	2057	652	0.063
	SMS−	1483	1455	1444	1437	1432	−51	0.879
	CAU	2131	1886	1787	1724	1677	−453	0.245
Moderate	SMS+	2505	2563	2587	2602	2613	107	0.833
	SMS−	2446	2579	2632	2666	2691	245	0.590
	CAU	2094	2500	2665	2769	2846	753	0.066
Vigorous	SMS+	2366	2415	2435	2448	2457	91	0.927
	SMS−	2609	2481	2429	2396	2371	−238	0.835
	CAU	3203	2916	2800	2726	2672	−531	0.660
Total physical activity	SMS+	5031	6207	6681	6984	7206	2175	**0.043**
	SMS−	5186	5516	5649	5734	5796	610	0.460
	CAU	5986	6029	6046	6057	6065	80	0.944
Sedentary min/week	Group	Baseline	3 months	6 months	9 months	12 months	Change	*p* value within
Total sitting	SMS+	2735	2265	2074	1953	1864	−871	**0.005**
	SMS−	2563	2397	2331	2288	2257	−306	0.183
	CAU	2559	2364	2285	2235	2198	−361	0.157

Note: Differences were tested with multilevel linear regression analyses combined with a bootstrap procedure in case of a non-normal distribution. Boldface indicates significant differences (*p* < 0.05). Abbreviations: IPAQ, international physical activity questionnaire; n, number; MET, metabolic equivalent of task; SMS+, lifestyle intervention with SMS support; SMS−, lifestyle intervention without SMS support; CAU, care as usual.

**Table 5:** Within-group changes in maximal cycle ergometer test outcomes from baseline to 12 months.

	Group	Baseline	3 Months	6 Months	9 Months	12 Months	Change	*p* Value within	*p* Value between
Max performance *n* (total)	SMS+	31 (46)	22 (25)	13 (15)	11 (12)	9 (11)	-	-	-
	SMS−	23 (40)	25 (29)	19 (22)	14 (18)	16 (19)	-	-	-
Peak load (watts)	SMS+	177	182	184	186	187	10	**0.016**	0.222
	SMS−	168	169	170	170	170	3	0.516	
% of achieved maximum HR *	SMS+	93	93	93	93	93	0	0.557	0.195
	SMS−	92	93	93	93	93	1	0.228	
Peak HR (BPM)	SMS+	175	174	173	173	173	−2	0.226	0.173
	SMS−	172	172	173	173	173	1	0.442	
mBorg	SMS+	6	6	6	6	6	0	0.688	0.552
	SMS−	6	6	6	6	6	0	0.647	

Note: Differences were tested with multilevel linear regression analyses for continuous variables, combined with a bootstrap procedure in case of a non-normal distribution. Boldface indicates significant differences (*p* < 0.05). * Achieved maximum HR was calculated with the Tanaka equation. Abbreviations: SMS+, lifestyle intervention with SMS support; SMS−, lifestyle intervention without SMS support; HR, heart rate; BPM, beats per minute; mBorg, modified Borg (rating of perceived exertion scale).

## Data Availability

The data presented in this study are available on request from the corresponding author. The data are not publicly available due to privacy restrictions.
